# Post-treatment Hematological Variations and the Role of Hemoglobin as a Predictor of Disease-free Survival in Stage 2 Breast Cancer Patients

**DOI:** 10.7759/cureus.7259

**Published:** 2020-03-13

**Authors:** Uzma Raza, Aminuddin Sheikh, Shah Nawaz Jamali, Mohsin Turab, Syeda Amber Zaidi, Haris Jawaid

**Affiliations:** 1 Biochemistry, Hamdard University, Karachi, PAK; 2 Pathology, Hamdard College of Medicine and Dentistry, Hamdard University, Karachi, PAK; 3 Pharmacology, Hamdard College of Medicine and Dentistry, Hamdard University, Karachi, PAK; 4 Pharmacology, Hamdard University, Karachi, PAK; 5 Oncology, Dow University of Health Sciences, Karachi, PAK

**Keywords:** anemia, breast cancer, disease free survival, hemoglobin

## Abstract

Objectives

The primary objective of this study was to determine post-treatment variations in the hematological profile of stage 2 breast cancer patients and investigate the influence of disease stage and treatment pattern on these changes. The secondary objective was to evaluate the role of post-treatment hemoglobin as a predictor of disease-free survival.

Methods

This prospective, observational study included 177 stage 2, female, breast cancer patients. Treatment included surgery, chemotherapy, radiotherapy, and hormonal (anti-estrogen) therapy. Patients were divided into treatment groups based on their histopathological features. Laboratory investigations, including hemoglobin and complete blood count, were carried out twice, first, at the initial cancer diagnosis and, second, eight weeks after completion of radiotherapy. The patients were followed for a period of four years and their disease-free survival was calculated.

Results

A significant post-treatment decrease in hemoglobin levels and red blood cell (RBC) count was observed in all patients except hormone receptor-positive disease stage 2A patients treated without chemotherapy. Total leukocyte counts were significantly decreased in all hormone receptor-negative patients, and significant neutropenia was observed in all stage 2 patients who received chemotherapy. The severity of anemia was observed to be significantly lower in stage 2A patients (without lymph node metastasis) as compared to stage 2B patients (with lymph node metastasis). Furthermore, no anemia was observed in hormone receptor-positive patients treated without chemotherapy, while moderate anemia was observed in hormone receptor-negative patients who received both pre and post-surgical chemotherapy. The post-treatment hemoglobin levels were found to be a significant predictor of disease-free survival in hormone receptor-positive patients (HR = 0.140, p= 0.000) and in patients of all disease stages except stage 2B (T3 N1 M0).

Conclusion

The incidence and severity of post-treatment anemia are low in patients treated with hormonal therapy and high in patients with lymph node metastasis. Higher post-treatment hemoglobin levels predict a longer duration of disease-free survival in hormone receptor-positive patients of disease stages 2A and 2B (T2).

## Introduction

Breast cancer is the most common cause of malignancy in women worldwide, with a reported 2019 incidence of 316,700 cases and mortality of 41,760 cases in the USA alone [1]. Studies have shown that while the incidence of breast cancer is higher in developed countries, mortality stays the highest in developing nations [2]. Early detection and management are the key factors necessary for a good prognosis in this disease. Efficient disease management involves a multidisciplinary team responsible for designing and administering complex treatment regimens involving combinations of multiple therapies (such as chemotherapy, surgery, radiation, etc) to effectively control and manage the disease [2]. Therefore, the absence of proper healthcare infrastructure and the general lack of awareness regarding breast cancer risk factors (including obesity after menopause, physical inactivity, nulliparity, and lack of breastfeeding) are postulated as key determinants of this high mortality [3].

There is considerable variation in the clinical manifestation and genotype of this disease, depending on a multitude of factors, including geography, race, and ethnicity [4]. This necessitates the consideration of each aforementioned factor in the course of research to develop more effective treatment and diagnostic strategies. The tumor, nodes, and metastasis (TNM) cancer staging system is used for diagnostic and treatment purposes and bases its classification on tumor size, metastasis and lymph node involvement [5-6].

As discussed, cancer treatment is a complex process entailing multiple treatment modalities, some of which (especially chemotherapy) have been associated with severe health implications. Anemia is a common consequence of such harsh treatments and has been well-documented in patients undergoing cancer therapy [7-9]. Furthermore, anemia pre-dating the disease has been associated with poor disease prognosis, poor response to treatment, and decreased disease-free survival (DFS) [10-11].

While research has unequivocally established that pre-treatment anemia leads to adverse disease outcomes, the role of post-treatment anemia as a predictor of disease-free survival still remains an area of interest, especially considering the variations in disease prognosis and management due to racial and geographic determinants. Therefore, this cohort study was designed to not only assess the prevalence and characteristics of post-treatment anemia in stage 2 breast cancer patients but also to evaluate the role of anemia (hemoglobin levels in particular) as a predictor of disease-free survival. The results of this study will help emphasize the importance of controlling anemia in such patients so as to improve their disease-free survival. Moreover, these findings can aid in the development of treatment guidelines for more effective disease management.

## Materials and methods

Study setting and design

This prospective cohort study was conducted at the oncology clinic of a local hospital in Karachi from 2011 - 2015, after obtaining ethical approval from the Board of Advanced Studies and Research (BASR), University of Karachi.

Patient population

A sample size of n = 156 patients was calculated using G*Power 3.1.9.4 (Cohen's d = 0.2, α-error = 0.05, power = 0.8), and the convenience sampling method was used for the collection of data.

The final study population included 177 non-pregnant, premenopausal, female breast cancer patients aged between 25 and 45 years, with infiltrating ductal carcinoma in just a single breast. The diagnosis of breast cancer had been confirmed via a histopathological evaluation (tumor biopsy), and the TNM system was used for cancer staging [5-6].

Patients included in the study were those in whom disease stage 2 had been confirmed and metastasis to different organs had been ruled out (via bone scan and whole-body ultrasound). Patients diagnosed with, or being treated for, any chronic disease (such as diabetes, cardiac disease, etc.) other than breast cancer were excluded from the study. Moreover, patients undergoing lumpectomy were also excluded from the study.

Table 1 shows the basic characteristics of each disease stage (tumor size, lymph node involvement, and metastasis). Five treatment groups were made for patients based on their disease stage and hormone receptor status. Patients were categorized as hormone receptor-positive if their tumors were positive for both estrogen and progesterone receptors. Furthermore, 10% of the lymph nodes recovered from stage 2B patients showed cancer metastasis (lymph node-positive).

**Table 1:** The basic group characteristics according to disease stage

Disease Stage	Tumor Size (cm)	No. of Patients (%)		Chemotherapy Treatment
		Hormone Receptor Positive (HRP)	Total	
2A (T2 N0 M0)	2.0-5.0	44 (72.1 %)	61	FAC (6 cycles):
				5-fluoroUracil = 500mg/m2
				Cyclophosphamide = 50mg/m2
				Adriamycin = 500mg/m2
2B (T2 N1 M0)	2.0-5.0	48 (57.1 %)	84	AC-T (4 cycles) followed by Paclitaxel (12 weekly cycles):
				Adriamycin = 60 mg/m2
				Cyclophosphamide = 600 mg/m2
				Paclitaxel (T) = 90 mg/m2
2B (T3 N1 M0)	>5.0	11 (34.4 %)	32	FAC (3 cycles pre surgery + 3 cycles post surgery):
				5-fluoroUracil = 500mg/m2
				Cyclophosphamide = 50mg/m2
				Adriamycin = 500mg/m2

The nature of the study was explained, and written informed consent was obtained from all the patients before their inclusion in the study.

Treatment options

Treatment options were provided by the oncologist based on tumor histopathology and past practice [12]. Mastectomy was the primary treatment option, followed by chemotherapy (with each cycle given at three-week intervals). In some cases, pre-post-surgery chemotherapy (FAC) was recommended for tumor shrinkage T3 (> 5 cm). Hormone receptor-positive (HRP) patients were given the option of elective chemotherapy, which they opted for based on their personal risk-benefit analysis once the details of the procedure had been explained to them. One week after completion of chemotherapy, radiotherapy (at the dose of 60 Gray) was prescribed for five days per week, lasting a total duration of six weeks. Parts exposed to radiotherapy included the tumor bed, axillary lymph nodes, and supraclavicular nodes. Patients with hormone receptor-positive tumors were treated with antiestrogens (Tamoxifen at 20 mg BD) starting immediately after treatment with radiotherapy and being continued further for five years.

Table 1 details the specifics of the chemotherapy regimen received by each group. Chemotherapy for lymph node-negative patients with disease stage 2A was six cycles of FAC (5-fluorouracil, cyclophosphamide, and adriamycin). Disease stage 2B lymph node-positive patients were prescribed a four-cycle course of AC-T followed by 12 cycles of weekly paclitaxel (T). For disease stage 2B patients with tumor size exceeding 5 cm, a total of six cycles of FAC (three cycles pre-surgery and three cycles post-surgery) was the prescribed treatment plan. All chemotherapy doses were adjusted according to patient height and bodyweight.

Measurement of outcomes

Blood Sampling and Analysis

Blood samples were collected twice; initially, at the time of disease diagnosis before the start of treatment and then at the end of the eighth week after the last radiotherapy dose. Hematological parameters measured included hemoglobin concentration and complete blood count (including leukocyte count, RBC count, platelet count, and percentages of lymphocytes and neutrophils). An automated hematology analyzer (Sysmex Corporation, Japan, code no. K-4500) was used for the estimation of these parameters [13].

Calculation of Disease-free Survival

The patients were followed for a period of 48 months after treatment, and disease-free survival (DFS) in weeks was calculated as the period between the day of full treatment completion and the day of recurrence of the first disease symptom or death.

Statistical analysis

All experimental data are expressed as mean ± SEM and all statistical analyses were conducted using IBM SPSS version 24 (IBM Corp., Armonk, NY). P < 0.05 was considered significant unless explicitly stated otherwise. Paired t-tests were used to statistically evaluate differences in the measured hematological parameters before and after treatment. Differences in the hematological profile before treatment were compared between groups via a two-way analysis of variance (ANOVA), and after treatment via a mixed ANOVA. Non-parametric tests (Somer’s d and Kruskal-Walis) were used to evaluate differences in anemia severity (according to the World Health Organization (WHO) criteria) between groups. Cox proportional hazards regression and Kaplan-Meier survival analysis were conducted to evaluate the predictive effect of post-treatment hemoglobin on disease-free survival according to treatment received and disease stage.

## Results

Post-treatment hematological variations

Data were stratified according to disease stage, and pre-treatment (Pre-T) and post-treatment (Post-T) hematological profiles were compared for each treatment option (Tables 2-4). Table 2 shows the hematological profile of patients without lymph node metastasis (LNM) at disease stage 2A (T2 N0 M0), all of whom underwent surgery. This group included 44 (72.1 %) HRP patients who received hormonal therapy in addition to radiotherapy with/without chemotherapy and 17 hormone receptor-negative (HRN) patients who exclusively underwent chemotherapy and radiotherapy after surgery. Only 38 patients belonging to the HRP group underwent all three: chemotherapy, radiotherapy, and hormonal therapy, subsequent to surgery (S/C/R/H). A statistically significant decrease in hemoglobin concentration (Pre-T = 12.02 ± 0.06 g/dL, Post-T = 11.52 ± 0.09, p<0.05), RBC count (Pre-T = 4.22 ± 0.07 x10^12^/l, Post-T=3.98 ± 0.04x10^12^/l), and neutrophil percentage (Pre-T = 65.92 ± 0.78, Post-T=51.92 ± 0.74, p<0.05) was observed in these patients after treatment. No statistically significant variation was observed in any hematological parameter of the remaining six HRP patients who only underwent radiotherapy after surgery, followed by hormonal therapy (S/R/H). With reference to the HRN patients (S/C/R), a statistically significant decrease was observed post-treatment in the mean hemoglobin concentration (Pre-T = 12.24 ± 0.04 g/dL, Post-T = 10.71 ± 0.09g/dL), along with the RBC count, neutrophil and lymphocyte percentages, and total lymphocyte count (TLC) (Table 2). As observed in the other two treatment groups, the post-treatment platelet count did not vary significantly.

**Table 2:** Hematological variations in stage 2A (T2 N0 M0) patients after treatment * represents a statistically significant difference of p< .05 compared to the pre-treatment values.

Treatment		Hemoglobin (g/dL)	Red Cell Count x10^12^/l	Total Leukocytes Count x10^ 9^/l	Platelets Count x10^ 9^/l	Neutrophils (%)	Lymphocyte (%)
Surgery, Chemotherapy, Radiotherapy, and Hormonal Therapy (n-38)	Pre-treatment	12.02 ± 0.06	4.22 ± 0.07	6.42 ± 0.33	318.39 ± 13.58	65.92 ± 0.78	29.63 ± 0.62
	Post-treatment	*11.52 ± 0.09	*3.98 ± 0.04	7.68 ± 0.28	298.58 ± 9.81	*51.92 ± 0.74	21.39 ± 0.58
Surgery, Chemotherapy, and Radiotherapy (n=17)	Pre-treatment	12.24 ± 0.04	4.19 ± 0.06	8.19 ± 0.30	303.12 ± 15.63	58.84 ± 1.24	30.60 ± 0.74
	Post-treatment	*10.71 ± 0.09	*3.72 ± 0.08	*5.36 ± 0.18	234.88 ± 9.66	*36.94 ± 1.14	*16.06 ± 1.01
Surgery, Radiotherapy, and Hormonal Therapy (n=6)	Pre-treatment	12.07 ± 0.10	4.13 ± 0.13	8.63 ± 0.69	297.67 ± 30.01	66.33 ± 2.80	29.83 ± 1.63
	Post-treatment	12.00 ± 0.04	4.10 ± 0.09	6.48 ± 0.68	332.67 ± 24.87	46.50 ± 3.66	18.83 ± 2.00

**Table 3:** Hematological variations in stage 2B (T2 N1 M0) patients after treatment * represents a statistically significant difference of p< .05 compared to the pre-treatment values

Treatments		Hemoglobin (g/dL)	Red Cell Count x10^12^/l	Total Leukocytes Count x10^9^/l	Platelets Count x10^ 9^/l	Neutrophils (%)	Lymphocyte (%)
Surgery + Chemotherapy + Hormonal Therapy With and Without Radiotherapy (n=39)	Pre-treatment	11.71 ± 0.07	4.34 ± 0.08	7.08 ± 0.33	312.21 ± 16.38	67.54 ± 1.80	27.03 ± 1.57
	Post-treatment	*11.42 ± 0.07	*3.94 ± 0.03	6.35 ± 0.16	307.85 ± 10.6	*50.15 ± 0.61	22.1 ± 0.68
Surgery + Chemotherapy and Radiotherapy (n=36)	Pre-treatment	11.74 ± 0.08	4.15 ± 0.05	8.57 ± 0.21	363.03 ± 14.75	63.98 ± 1.63	31.57 ± 0.45
	Post-treatment	*10.56 ± 0.06	*3.77 ± 0.05	*5.30 ± 0.1	239.33 ± 5.53	*37.39 ± 0.87	*16.06 ± 0.61
Surgery + Radiotherapy and Hormonal Therapy (n=9)	Pre-treatment	11.93 ± 0.03	4.13 ± 0.07	7.78 ± 0.41	301.56 ± 28.37	63.11 ± 2.29	30.67 ± 0.68
	Post-treatment	*11.64 ± 0.13	3.89 ± 0.11	6.44 ± 0.63	292.44 ± 20.8	*44.33 ± 2.28	*18.67 ± 1.38

**Table 4:** Hematological variations in stage 2B (T3 N1 M0) patients after treatment * represents a statistically significant difference of p< .05 compared to the pre-treatment values

Treatments		Hemoglobin (g/dL)	Red Cell Count x10^12^/l	Total Leukocytes Count x10^ 9^/l	Platelets Count x10^9^/l	Neutrophils (%)	Lymphocyte (%)
Chemotherapy + Surgery + Chemotherapy + Radiotherapy and Hormonal Therapy (n=11)	Pre-treatment	11.84 ± 0.09	4.15 ± 0.06	8.17 ± 0.46	332.45 ± 29.91	64.18 ± 2.32	30.00 ± 1.21
	Post-treatment	*10.76 ± 0.08	*3.97 ± 0.08	6.77 ± 0.62	273.45 ± 23.28	*40.27 ± 0.45	*20.18 ± 0.97
Chemotherapy + Surgery + Chemotherapy with and without Radiotherapy (n=21)	Pre-treatment	11.67 ± 0.09	3.98 ± 0.07	8.52 ± 0.32	343.86 ± 11.94	64.62 ± 2.12	30.71 ± 0.87
	Post-treatment	*10.32 ± 0.08	*3.89 ± 0.06	*5.47 ± 0.11	243.38 ± 5.63	*36.48 ± 1.30	*17.33 ± 0.92

A total of 84 patients with LNM and disease stage 2B (T2 N1 M0) were included in the study (Table 3), all of whom underwent surgery. The HRP patients (48, 52.5 %) were treated with hormonal therapy along with either radiotherapy exclusively or in combination with chemotherapy. The remaining 36 HRN patients received post-surgery chemotherapy along with radiotherapy (S/C/R). The hematological profiles of 39 of the HRP patients who received chemotherapy with or without radiotherapy (S/C/R/H), revealed a significant decrease in post-treatment hemoglobin concentration (Pre-T = 11.71 ± 0.07 g/dL, Post-T = 11.30 ± 0.06 g/dL, p<0.05), RBC count (Pre-T = 4.34 ± 0.08 x10^12^/l, Post-T=3.94 ± 0.03x10^12^/l), and neutrophil percentage (Pre-T = 67.54 ± 1.8 %, Post-T = 50.15 ± 0.61 %, p<0.05) (Table 3). In the other nine HRP patients who just received radiotherapy in conjunction with hormonal therapy after surgery (S/R/H), statistically significant differences were observed in mean post-treatment hemoglobin (Pre-T = 11.93 ± 0.03 g/dL, Post-T = 11.64 ± 0.13 g/dL), lymphocyte percentage (Pre-T = 30.67 ± 0.68 %, Post-T = 18.67 ± 1.38 %), and neutrophil percentage (Pre-T = 63.11 ± 2.29 %, Post-T = 44.33 ± 2.28 %). The profile of the 36 HRN patients who received post-surgical chemotherapy and radiotherapy exclusively revealed a statistically significant decrease in all measured hematological parameters except for the platelet counts (Table 3).

Table 4 shows that 32 patients at disease stage 2B (T3N1M0), with axillary lymph node metastasis and tumor size > 5 cm, were included in the study. There were 11 (34.3%) HRP patients who received both pre- and post-surgery chemotherapy along with post-surgical radio and hormonal therapies (C/S/C/R/H). Hematological findings in these patients revealed significantly marked decreases in post-treatment hemoglobin levels (Pre-T = 11.84 ± 0.09 g/dL, Post-T = 10.76 ± 0.08 g/dL), RBC count (Pre-T = 4.15 ± 0.06x10^12^/l, Post-T=3.97 ± 0.08x10^12^/l), neutrophil percentage (Pre-T = 64.18 ± 2.32 %, Post-T = 30.00 ± 1.21%), and lymphocyte percentage (Pre-T = 40.27 ± 0.45 %, Post-T = 20.18 ± 0.97%) (Table 4). The remaining 21 (65.6%) HRN patients received pre- and post-chemotherapy without hormonal therapy, with and without radiotherapy (C/S/C/R). Their profiles showed a significantly decreased post-treatment TLC (Pre-T = 8.52 ± 0.32 x 109/L, Post-T= 5.47 ± 0.11 x 109/L), decreased post-treatment hemoglobin levels (Pre-T = 11.67 ± 0.09 g/dL, Post-T = 10.15 ± 0.04 g/dL), RBC count (Pre-T = 3.98 ± 0.07x10^12^/l, Post-T=3.89 ± 0.06x10^12^/l), neutrophil percentage (Pre-T = 64.62 ± 2.12 %, Post-T = 36.48 ± 1.3 %), and lymphocyte percentage (Pre-T = 30.71 ± 0.87 %, Post-T = 17.33 ± 0.92 %).

Hematological variations between groups

A two-way ANOVA was conducted to compare the effect of disease stage and treatment on pretreatment hemoglobin levels. Analysis revealed that hemoglobin levels varied significantly only with disease stage (F=13.58, p=0.000). Post-hoc comparisons showed that while mean pretreatment hemoglobin levels were significantly higher in stage 2A patients as compared to the other groups, they did not differ significantly between stage 2B and 2B(T3) patients (Table 5).

**Table 5:** Differences in pre-treatment hemoglobin between disease stages * a significance of p<0.05 was observed ** a significance of p<0.01 was observed

Pairs by Disease Stage	Mean Hemoglobin Difference/gdL⁻¹ (± SEM)	Significance
2A - 2B	0.34 ± 0.07	*0.000
2A - 2B(T3)	0.35 ± 0.09	*0.000
2B - 2B(T3)	0.02 ± 0.08	0.973

A mixed-design ANOVA on hemoglobin levels revealed a significant interaction between time and treatment groups (F=33.89, p=0.000). Since Leven’s test of homogeneity of variances was violated, p<0.001 was considered significant for this analysis. Although there were no significant differences between groups at baseline, significant variation was observed post-treatment. The post-treatment hemoglobin level of patients of S/R/H and S/C/R/H was significantly higher than patients who received S/C/R, C/S/C/R/H, and C/S/C/R (Table 6). No significant variation in post-treatment hemoglobin level was observed between groups C/S/C/R, S/C/R, and C/S/C/R/H, nor between groups S/R/H and S/C/R/H.

**Table 6:** Post-treatment hemoglobin variation between treatment groups *p<0.05 considered statistically significant **p<0.001 considered statistically significant

Pairs by Treatment Group	Mean Hemoglobin Difference/gdL⁻¹ (± SEM)	Significance
C/S/C/R - C/S/C/R/H	-0.44 ± 0.16	0.052
C/S/C/R - S/C/R	-0.29 ± 0.11	0.083
C/S/C/R - S/C/R/H	-1.15 ± 0.1	**0.000
C/S/C/R - S/R/H	-1.46 ± 0.14	**0.000
C/S/C/R/H - S/C/R	0.15 ± 0.14	1.000
C/S/C/R/H - S/C/R/H	-0.71 ± 0.14	**0.000
C/S/C/R/H - S/R/H	-1.02 ± 0.17	**0.000
S/C/R - S/C/R/H	-0.86 ± 0.08	**0.000
S/C/R - S/R/H	-1.18 ± 0.12	**0.000
S/C/R/H - S/R/H	-0.32 ± 0.12	0.082

Variation in post-treatment anemia

The WHO criteria for the grading of anemia were used to group post-treatment hemoglobin values into one of four categories: normal, mild, moderate, or severe [14]. Somer’s d test found a significant positive correlation between disease stage and severity of anemia (d=0.400, p=0.000). Table 7 shows that as disease stage progresses from 2A to 2B(T3), the percentage of patients with moderate anemia increases (from 27.9% in 2A to 93.8% in 2BT3) while the corresponding percentage for normal hemoglobin decreases (from 26.2% in 2A to 0% in 2BT3). Furthermore, the Kruskal-Wallis test showed a significant association between treatment group and anemia (χ2 = 123.241, p=0.000). Pairwise comparisons revealed significant differences in anemia distribution between S/R/H and S/C/R, C/S/C/R/H, C/S/C/R; and between S/C/R/H and S/C/R, C/S/C/R/H, C/S/C/R. The two hormonal therapy treatments (S/C/R/H and S/R/H) had comparatively more patients with normal hemoglobin (23.4% and 46.7%, respectively) and fewer patients with moderate anemia (10.4% and 0%, respectively) (Table 7). Compared to this, 100% of C/S/C/R patients were suffering from moderate anemia, followed by S/C/R (94.3%) and C/S/C/R/H (81.8%).

**Table 7:** Anemia categorization in patients according to treatment group and disease stage WHO: World Health Organization

		WHO Anemia Category			Total
		Normal (Hb > 12.0 g/dL)	Mild (Hb = 11.0-11.9 g/dL)	Moderate (Hb = 8.0-10.9 g/dL)	
Treatment	C/S/C/R	0 (0%)	0 (0%)	21 (100%)	21
	C/S/C/R/H	0 (0%)	2 (18.2%)	9 (81.8%)	11
	S/C/R	0 (0%)	3 (5.7%)	50 (94.3%)	53
	S/C/R/H	18 (23.4%)	51 (66.2%)	8 (10.4%)	77
	S/R/H	7 (46.7%)	8 (53.3%)	0 (0%)	15
Stage	2A	16 (26.2%)	28 (45.9%)	17 (27.9%)	61
	2B	9 (10.7%)	34 (40.5%)	41 (48.8%)	84
	2B (T3)	0 (0%)	2 (6.3%)	30 (93.8%)	32

Impact of post-treatment hemoglobin on DFS

The use of the Cox proportional hazards-regression model showed an overall significant predictive effect of post-treatment hemoglobin on patient disease-free survival (DFS) (HR = 0.263, p=0.0.000). Treatment-wise analysis revealed that hemoglobin was significantly associated with longer DFS in hormone receptor-positive patients (HR=0.140, p=0.000). Significant positive associations between post-treatment hemoglobin in both S/R/H (HR = 0.03, p=0.003) and S/C/R/H (HR=0.15, p=0.000) were observed (Table 8). It can be interpreted that in patients who underwent SRH, a 1 g/dL increase in post-treatment hemoglobin corresponded to a 97% increase in DFS. When analyzed by disease stage, a significant association of post-treatment hemoglobin with DFS was only observed in stage 2A (HR = 0.12, p=0.000) and 2B (T2) (HR = 0.19, p=0.000) patients.

**Table 8:** Cox-regression analysis for post-treatment hemoglobin and disease-free survival * p<0.05 is considered statistically significant ** p<0.01 is considered statistically significant

		HR (95% CI)	Significance
Hormone receptor status	Negative	0.16 (0.34-1.13)	0.115
	Positive	0.14 (0.09-0.22)	**0.000
Treatment Group	C/S/C/R	1.28 (0.37-4.39)	0.696
	C/S/C/R/H	0.11 (0.01-1.37)	0.086
	S/C/R	0.59 (0.28-1.25)	0.170
	S/C/R/H	0.15 (0.09-0.27)	**0.000
	S/R/H	0.03 (0.00-0.32)	0.003
Stage	2A (T2 N0 M0)	0.12 (0.06-0.22)	**0.000
	2B (T2 N1 M0)	0.19 (0.13-0.30)	**0.000
	2B (T3 N1 M0)	1.20 (0.49-2.97)	0.690

**Figure 1:**
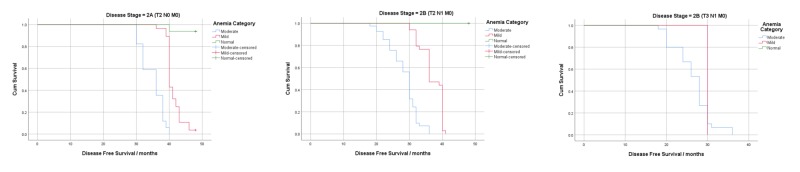
Survival analysis using post-treatment anemia according to disease stage

**Figure 2:**
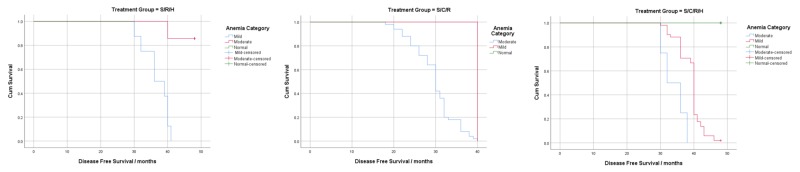
Survival analysis using post-treatment anemia according to treatment pattern (stages 2A and 2B-T2)

**Figure 3:**
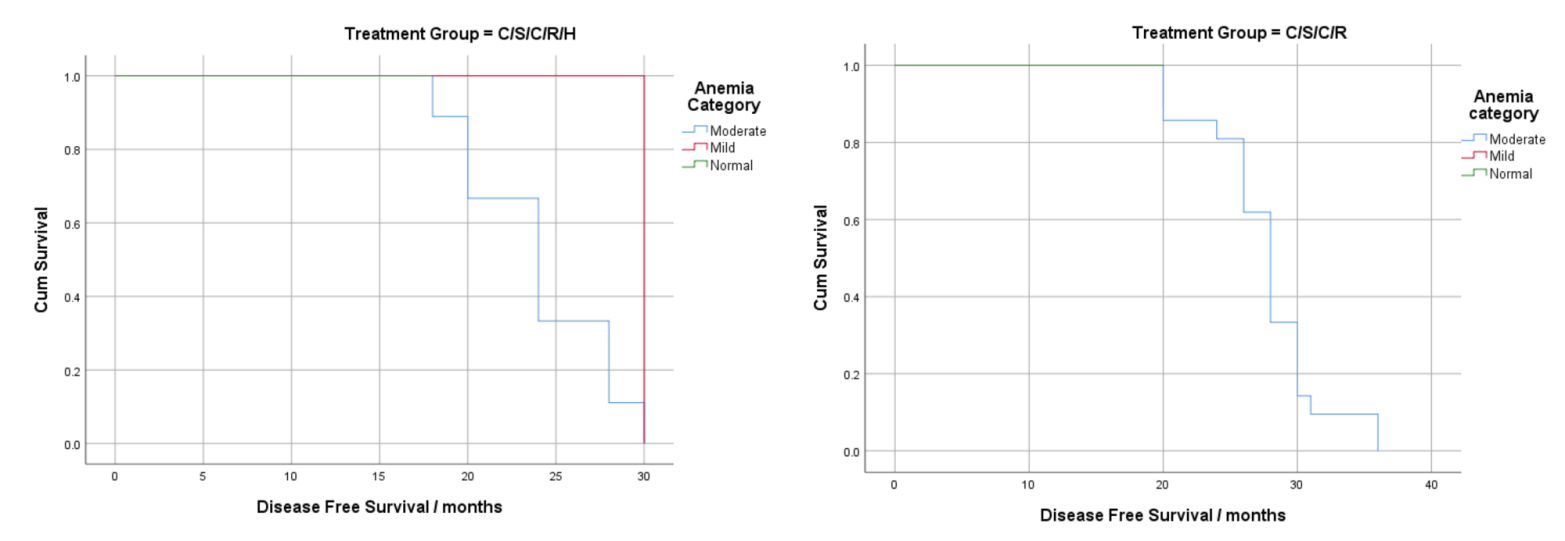
Survival analysis using post-treatment anemia according to treatment pattern (stage 2B-T3)

Kaplan-Meier curves were constructed to visualize the effect of post-treatment anemia on DFS (Figures 1-3). Log-rank tests were significant for all treatment groups (except CSCRH, p=0.051) and for all disease stages except stage 2B(T3) (p=0.301) and showed that the overall DFS decreased as the severity of anemia increased.

## Discussion

This study revealed that the patients’ hematological profile not only varies significantly after treatment but is also influenced by the pattern of treatment and disease stage. Additionally, significant differences in patients’ pretreatment hemoglobin levels were observed between disease stages (d=0.400, p=0.000) but understandably not between treatment groups. The decrease in hemoglobin levels with advancing disease was significant in cases where lymph node metastasis had occurred (between stages 2A and 2B) but not where tumor size had increased (between stages 2B-T2 and 2B-T3) (Table 5). Anemia in cancers is well-documented and is attributed largely to the release of inflammatory cytokines (interleukin 6 (IL-6) in particular) [15]. These inflammatory mediators are secreted by both immune cells and cancer cells and decrease the RBC count and hemoglobin levels via a range of mechanisms. Such mechanisms include but are not limited to the impairment of iron metabolism, increased hemolysis (via redox reactions), decreased direct erythropoiesis, and decreased erythropoietin production [15-16]. A major consequence of IL-6 activity is the overproduction of hepcidin, which in turn decreases iron utilization and absorption from the gut, resulting in anemia and low hemoglobin levels [16]. Therefore, an increase in anemia with disease progression (especially lymph node metastasis) can be credited to the increased release of inflammatory cytokines due to the involvement of more immune and tumor cells.

A statistically significant post-treatment decrease was found in the mean hemoglobin, RBC count, and leukocyte counts of patients treated without hormonal therapy (Tables 2-4). Breast cancer treatment is complex and usually entails multiple therapies, including surgical techniques, radiotherapy, chemotherapy, and hormonal (anti-estrogen) therapy [17]. Hemoglobin levels were consistently decreased in all disease stages and treatment groups, except in stage 2A patients receiving S/R/H (Pre-T= 12.07 ± 0.10 g/dL, Post-T=12.00 ± 0.04g/dL, p>0.05). The RBC counts were significantly lower in all cases except for patients who received hormonal therapy without chemotherapy (Tables 2-3). These findings suggest that anti-estrogen hormonal therapy may play a protective role in breast cancer patients against anemia. A study reported that approximately 70% of tumor cells in breast cancer patients have estrogen receptors, and anti-estrogen drugs can improve disease prognosis by targeting these cells [18]. Furthermore, several studies have associated chemotherapy with adverse hematological outcomes in such patients [7-9,19-20]. Chemotherapeutic agents, such as platinum salts, anthracycline, and gemcitabine, have been associated with post-treatment anemia. The proposed mechanism of action for this is the inhibition of hematopoiesis brought on by decreased erythropoietin levels secondary to renal dysfunction [20]. This explains the significant decrease in both hemoglobin and RBC in patients who received chemotherapy, especially in those without hormonal therapy. Moreover, the myelosuppressive effect of chemotherapy has been well-documented and is responsible for the progression of anemia with each cycle of chemotherapy [19]. Radiotherapy has also been implicated to some extent as a cause of post-treatment anemia via hemolysis, accomplished by an increase in RBC membrane fragility due to lipid oxidation [21]. This justifies that anemia, when graded according to the WHO criteria, was the most severe in stage 2B(T3) HRN patients (100% patients were moderately anemic) while being the least severe in HRP patients of stage 2A and 2B (Table 7). Concerning leukocyte counts, chemotherapy has been well-associated with leukopenia, especially in the treatment of gynecological malignancies [22]. Neutropenia is a well-documented, dose-limiting side effect of such therapies and explains the leukopenia observed in all treatment groups that included chemotherapy (Tables 2-5) [23]. Research has also shown that hormonal therapy (tamoxifen) can help mitigate leukopenia due to chemotherapy and radiotherapy and this would explain the increased severity of leukopenia in HRN patients [24]. 

Post-treatment hemoglobin significantly affected DFS in disease stages 2A and 2B(T2) and in hormone receptor-positive patients (excluding stage 2B(T3)) (Table 8). Our analysis showed that a 1 gm/dL increase in the post-treatment hemoglobin levels of HRP patients reduced recurrence and mortality by 86% (HR=0.14, p=0.000). Our findings are, therefore, consistent with previously conducted research, which associates a good disease prognosis with hormonal therapy [18]. It can be inferred from this that in HRN patients, other factors may have a more prominent effect on DFS than anemia. Furthermore, in the advanced disease stage (2B (T3)), post-treatment hemoglobin was not associated with DFS (HR = 1.20, p=0.690). The Kaplan-Meier curves for post-treatment hemoglobin stratified by disease stage show an overall decrease in cumulative survival and shorter DFS, as the severity of anemia worsens (Figure 1). These findings are consistent with the results of a study conducted by Choi et al., which reported that hemoglobin maintenance only significantly increased survival in patients without lymph node metastasis [25]. 

Study limitations

The findings of this study, however, must be considered keeping the following limitations in mind. Firstly, when stratified according to the pattern of treatment, certain treatment groups had fewer than 10 participants. Therefore, the power of statistical analysis involving these groups would be low and this should be taken into consideration. Secondly, the use of convenience sampling and the collection of data from a single hospital introduces the possibility of the cohort not being a true representative of the actual patient population. Finally, the follow-up period for our patients after treatment was relatively short (48 months), as more long-term follow-up was both difficult and unfeasible in our setup. It is our recommendation that more multi-center longitudinal studies be conducted on much larger patient populations with longer follow-up periods (for 5-10 years) in order to ascertain the validity of these findings.

## Conclusions

Post-treatment hematological variations were largely attributed to the treatment pattern rather than the disease stage. However, the incidence and severity of post-treatment anemia increased with advancing disease stage (lymph node metastasis and/or increase in tumor size). Treatment options also influenced anemia, being most severe in treatment including chemotherapy without hormonal therapy. This study suggests that hormonal therapy protects against while chemotherapy exacerbates the post-treatment decrease in hemoglobin, RBC count, and leukocyte count. Post-treatment hemoglobin was determined to be a significant predictor of disease-free survival in only HRP patients of disease stages 2A and 2B(T2).
